# Epigenetic Regulation of the N-Terminal Truncated Isoform of Matrix Metalloproteinase-2 (NTT-MMP-2) and Its Presence in Renal and Cardiac Diseases

**DOI:** 10.3389/fgene.2021.637148

**Published:** 2021-02-25

**Authors:** Juliana de Oliveira Cruz, Alessandra O. Silva, Jessyca M. Ribeiro, Marcelo R. Luizon, Carla S. Ceron

**Affiliations:** ^1^Graduate Program in Genetics, Institute of Biological Sciences, Federal University of Minas Gerais, Belo Horizonte, Brazil; ^2^Department of Genetics, Ecology and Evolution, Institute of Biological Sciences, Federal University of Minas Gerais, Belo Horizonte, Brazil; ^3^Department of Food and Drugs, Faculty of Pharmaceutical Sciences, Federal University of Alfenas, Alfenas, Brazil; ^4^Department of Biological Sciences, Institute of Exact and Biological Sciences, Federal University of Ouro Preto, Ouro Preto, Brazil

**Keywords:** acute kidney injury, alternative promoter, chronic kidney disease, histone modifications, matrix metalloproteinase-2, mitochondria, epigenetics (DNA methylation), epigenomics

## Abstract

Several clinical and experimental studies have documented a compelling and critical role for the full-length matrix metalloproteinase-2 (FL-MMP-2) in ischemic renal injury, progressive renal fibrosis, and diabetic nephropathy. A novel N-terminal truncated isoform of MMP-2 (NTT-MMP-2) was recently discovered, which is induced by hypoxia and oxidative stress by the activation of a latent promoter located in the first intron of the *MMP2* gene. This NTT-MMP-2 isoform is enzymatically active but remains intracellular in or near the mitochondria. In this perspective article, we first present the findings about the discovery of the NTT-MMP-2 isoform, and its functional and structural differences as compared with the FL-MMP-2 isoform. Based on publicly available epigenomics data from the Encyclopedia of DNA Elements (ENCODE) project, we provide insights into the epigenetic regulation of the latent promoter located in the first intron of the *MMP2* gene, which support the activation of the NTT-MMP-2 isoform. We then focus on its functional assessment by covering the alterations found in the kidney of transgenic mice expressing the NTT-MMP-2 isoform. Next, we highlight recent findings regarding the presence of the NTT-MMP-2 isoform in renal dysfunction, in kidney and cardiac diseases, including damage observed in aging, acute ischemia-reperfusion injury (IRI), chronic kidney disease, diabetic nephropathy, and human renal transplants with delayed graft function. Finally, we briefly discuss how our insights may guide further experimental and clinical studies that are needed to elucidate the underlying mechanisms and the role of the NTT-MMP-2 isoform in renal dysfunction, which may help to establish it as a potential therapeutic target in kidney diseases.

## Introduction

Matrix metalloproteinases (MMPs) are a large family of zinc-containing endopeptidases that participate in multiple cellular processes beyond extracellular matrix (ECM) remodeling and in kidney diseases (Tan and Liu, 2012; Parrish, 2017). Regarding the MMPs known as gelatinases, the full-length MMP-2 (FL-MMP-2) is synthesized and was originally thought to be only secreted, but later it was found to be only inefficiently targeted to the secretory pathway (Ali et al., 2012). The FL-MMP-2 can be activated by extracellular proteolysis and intracellularly by direct chemical modification by peroxynitrite/oxidative stress (Viappiani et al., 2009; Kandasamy et al., 2010; Sariahmetoglu et al., 2012).

In the renal ECM, FL-MMP-2 has a role in the regulated turnover of the tubular epithelial basement membrane (Cheng et al., 2006). However, enhanced FL-MMP-2 synthesis distorts the basement membrane architecture and results in progressive renal injury (Cheng et al., 2006), cardiac remodeling and contractile dysfunction (Jacob-Ferreira and Schulz, 2013). The regulation and role of FL-MMP-2 have been extensively studied in both kidney (Tan and Liu, 2012; Dimas et al., 2017; Mansour et al., 2017; Narula et al., 2018) and cardiac injuries (Coker et al., 1999; Wang et al., 2002; Sawicki et al., 2005; Sung et al., 2007; Ali et al., 2010). Besides ECM proteins, FL-MMP-2 also cleaves vasoactive peptides, chemokines, and intracellular sarcomere and nuclear proteins (Fernandez-Patron et al., 1999, 2000; Martinez et al., 2004; Denney et al., 2009; Jacob-Ferreira and Schulz, 2013; DeCoux et al., 2014).

A novel N-terminal truncated isoform of MMP-2 (NTT-MMP-2) was recently discovered, which is induced by hypoxia and oxidative stress by the activation of a latent promoter in the first intron of the *MMP2* gene, thereby generating a 5′-truncated mRNA transcript (Lovett et al., 2012). This NTT-MMP-2 isoform is enzymatically active but lacks the secretory sequence and the inhibitory propeptide, remains intracellular in the mitochondria, triggers mitochondrial-nuclear stress signaling *via* nuclear factor-κβ (NF-κβ) and nuclear factor of activated T cells (NFAT), and induces innate immune response genes (Lovett et al., 2012).

This perspective presents the findings about the discovery of the NTT-MMP-2 isoform, its functional and structural differences as compared with the FL-MMP-2 isoform, the alterations in the kidney of transgenic mice expressing the NTT-MMP-2 isoform, insights into the epigenetic regulation of the latent promoter located in the first intron of *MMP2* gene that support the activation of the NTT-MMP-2 isoform, and highlights the role of the NTT-MMP-2 isoform in renal and cardiac diseases.

## The FL-MMP-2 and the Novel NTT-MMP-2 Isoform

The structure of the 68 kDa FL-MMP-2 is a short N-terminal signal sequence for secretory vesicle processing, a propeptide domain responsible for the enzyme latency, a highly conserved zinc-binding catalytic domain and hemopexin and fibronectin domains, which binds to ECM substrates (Turck et al., 1996; Morgunova et al., 1999). The FL-MMP-2 latency is maintained by the presence of a cysteine residue of the prodomain extended along the catalytic site, the “cysteine-switch” mechanism. The mRNA transcript of FL-MMP-2 is translated, and a portion of enzymatically inactive full-length protein is secreted by vesicles to the extracellular space, where occurs the proteolytic activation of the latent MMP-2 protein by other MMPs, ending in an active 62 kDa MMP-2 after cleavage of the inhibitory prodomain. In the ECM, the active enzyme degrades ECM components, such as collagen IV, laminin, and elastin (Turck et al., 1996; Morgunova et al., 1999).

The intracellular MMP-2 was first observed distributed in a pattern consistent with sarcomeric and sarcolemmal in cardiomyocytes (Coker et al., 1999), and the cleavage of sarcomeric troponin I by the 68 kDa FL-MMP-2 following acute ischemia-reperfusion injury (IRI) of the heart was later reported (Wang et al., 2002). Next, myosin light chain-1 (Sawicki et al., 2005), α-actinin (Sung et al., 2007), and titin (Ali et al., 2010) were reported as intracellular targets of the FL-MMP-2 in cardiomyocytes, leading to impaired heart contractility. This intracellular 68 kDa FL-MMP-2 is able to escape the secretory pathway (Ali et al., 2012), and is activated by the cysteine-switch opening by reactive oxygen species and peroxynitrite (Viappiani et al., 2009), and its activity was shown to be further modulated by its phosphorylation in cardiomyocytes (Sariahmetoglu et al., 2007, 2012).

**FIGURE 1:**
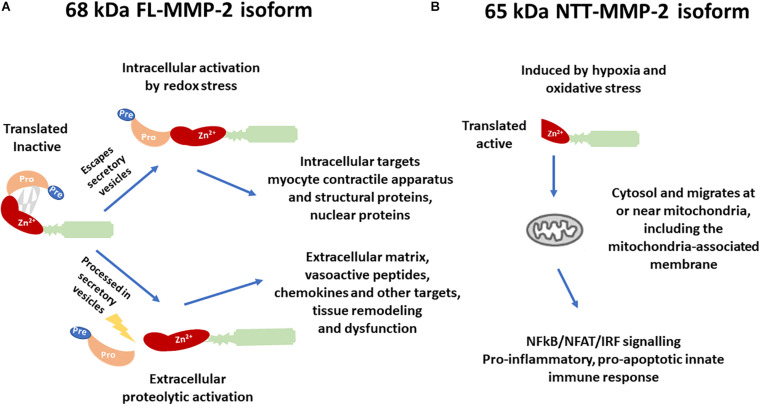
Differences in the structure, activation, and biological targets/outcomes between **(A)** the 68 kDa full-length isoform of MMP-2 (FL-MMP-2) and **(B)** the 65 kDa N-terminal truncated isoform of MMP-2 (NTT-MMP-2).

Regarding kidney diseases, most of the studies focused on the extracellular or intracellular roles of the FL-MMP-2, mainly its deleterious action in the tubular basement membrane (Cheng et al., 2006). A novel intracellular MMP-2 isoform with 65 kDa (NTT-MMP-2) was recently discovered, which is generated by the activation of an alternate promoter in the first intron of the *MMP2* gene (Lovett et al., 2012). This novel isoform was first observed in the hearts of both FL-MMP-2 transgenic mice and aging wild-type mice (Lovett et al., 2012). Cardiomyoblasts submitted to mitochondrial stress generated with transient inhibition of oxidative phosphorylation induced the NTT-MMP-2 isoform expression on mitochondria-enriched cell fractions (Lovett et al., 2012). The NTT-MMP-2 is functionally and structurally distinct from the FL-MMP-2 (Figure 1).

While the FL-MMP-2 is present in the cytoplasm or ECM, and in cells of control conditions, the NTT-MMP-2 is intracellular, predominantly located at or near the mitochondria, and its transcription is induced only in conditions of tissue damage (Lovett et al., 2012). Transfection of the NTT-MMP-2 cDNA in cardiomyoblasts resulted in increased luciferase reporter activity for NF-kβ, NFAT, and response elements for interferon regulatory factors (IRFs) and induced innate immune response transcription factors and chemokines/cytokines, thereby activating a proinflammatory, pro-apoptotic innate immune response (Lovett et al., 2012).

## Epigenetic Regulation of the NTT-MMP-2 Isoform Expression

The transcriptional start site for the FL-MMP-2 isoform is located in the 5′ flanking region of *MMP2*, and transcription from this site encodes the FL-MMP-2 beginning at M1 amino acid in the first exon of the *MMP2* gene (Lovett et al., 2012). Transcription of NTT-MMP-2 starts with activation of a latent promoter induced by hypoxia and oxidative stress, in an alternate transcriptional start site located at the 3′ end of the first intron of the *MMP2*, which encodes the NTT-MMP-2 isoform beginning at M77 amino acid located within the second exon of *MMP2* (Lovett et al., 2012).

**FIGURE 2:**
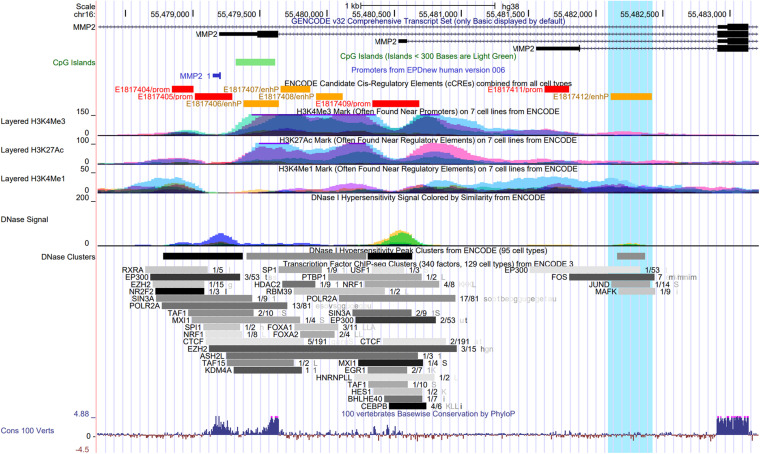
UCSC Genome Browser view of the promoter and the first intron of the *MMP2* gene showing the overlap with several epigenomics data from the ENCODE Project, including the ENCODE registry of candidate cis-regulatory elements (cCREs), DNase I hypersensitivity clusters, ChIP-seq data for three histone marks on seven cell lines, and Transcription Factor ChIP-seq clusters. The transcriptional start site in the promoter region of *MMP2* gene is indicated by the Eukaryotic Promoter Database (EPDnew). Highlighted (in light blue) is a region with an enhancer-like signature (E1817412/enhP) identified by the ENCODE cCREs in the first intron of *MMP2*, which overlaps with a DNase I hypersensitivity cluster, and with ENCODE ChIP-seq data for the histone modification H3K4me1, for the histone acetyltransferase EP300, and for the hypoxia-induced transcription factors FOS, JUND, and MAFK.

Figure 2 shows the promoter region of the *MMP2* with the transcriptional start site as indicated by the Eukaryotic Promoter Database (Dreos et al., 2013) and highlights the overlap of the first intron with several epigenomics data from the Encyclopedia of DNA Elements (ENCODE) (Consortium, 2012) and GENCODE consortium (Frankish et al., 2019), including ENCODE registry of candidate *cis*-regulatory elements (cCREs), DNase I hypersensitivity clusters (Thurman et al., 2012), chromatin immunoprecipitation-sequencing (ChIP-seq) data for histone marks, and transcription factor ChIP-seq clusters (Consortium, 2012). This approach using epigenomics data to identify gene regulatory regions was recently performed elsewhere (Linhares et al., 2020).

## Histone Modifications and the NTT-MMP-2 Isoform Expression

The trimethylation of histone H3 on lysine 4 (H3K4me3) is associated with promoters (Rosenbloom et al., 2012). The acetylation of histone H3 on lysine 27 (H3K27ac) is often found near active regulatory elements such as enhancers and distinguishes active from poised enhancers containing the monomethylation of histone H3 on lysine 4 (H3K4me1) alone (Creyghton et al., 2010).

H3K4me1 is commonly associated with distal enhancers, but it is also present at promoter regions proximal to transcriptional start site (Cheng et al., 2014; Bae and Lesch, 2020). Noteworthy, H3K4me1 peak density was recently examined around promoters in human and mouse germ cells (Bae and Lesch, 2020). H3K4me1 was found to exhibit either a bimodal pattern at active promoters, where it flanks H3K4me3, or a unimodal pattern at poised promoters, where it coincides with both H3K4me3 and trimethylation at lysine 27 of histone H3 (H3K27me3). H3K4me1 distribution was proposed as a key feature of the poised epigenetic state and poising at promoters (Bae and Lesch, 2020).

Interestingly, a region with enhancer-like signature (E1817412/enhP) identified by the ENCODE cCREs in the first intron of the *MMP2* gene overlaps with a DNase I hypersensitivity cluster, and with ENCODE ChIP-seq data for the histone modification H3K4me1, for the histone acetyltransferase EP300, and for the transcription factors FOS, JUND, and MAFK, which are known to cooperate in hypoxia-induced gene transcription (Figure 2). Hypoxic conditions were shown to induce the transcriptional activation of c-fos in HeLa cells (Muller et al., 1997). Notably, c-Jun was shown to functionally cooperate with hypoxia-inducible factor 1 (HIF-1) transcriptional activity in different cell types (Alfranca et al., 2002). In this context, the small Maf protein, MafG, possess a basic leucine zipper domain that is required for homodimer or heterodimer complex formation with other transcription factors. MafG was shown to interact with HIF-1α, a key factor in hypoxic response, and it was suggested to regulate the hypoxic response by detaining HIF-1α in the nucleus (Ueda et al., 2008). These data further support the presence of the poised promoter located in the first intron of the *MMP2* gene and suggest that it may be affected by a putative enhancer element activated by the binding of well-known hypoxia-induced transcription factors in the activation of the NTT-MMP-2 expression. However, this hypothesis remains to be tested.

## DNA Methylation and the NTT-MMP-2 Isoform Expression

DNA methylation consists of the addition of the methyl group in cytosines followed by guanines, named CpG dinucleotides. CpG islands are regions enriched in CpG dinucleotides that are normally located in gene promoters and that are implicated in the regulation of gene expression (Gardiner-Garden and Frommer, 1987; Jones, 2012). ENCODE data show a CpG island located in the promoter/exon 1 of the *MMP2* gene (Figure 2), which are not methylated in most of the ENCODE cell lines. However, there is no CpG methylation data by Methyl 450K Bead Arrays from ENCODE, which overlap with the latent promoter region in the intron 1 of the *MMP2* gene (Supplementary Figure 1).

DNA methylation represses gene transcription when located in promoter regions and activates transcription when located in gene body (Jones, 2012). Methylation pattern is known to differ between promoters and alternative promoters in the same gene and among different tissues, indicating a dynamic physiological change in DNA methylation. Thus, DNA methylation may have a significant role in the differential use of alternative promoters, which may be related to the functional differentiation of promoters with or without CpG islands (Cheong et al., 2006). Moreover, intragenic DNA methylation has a major role in regulating alternative promoters in gene bodies (Maunakea et al., 2010). While there are no DNA methylation data from ENCODE for the intron 1 of the *MMP2* gene, there are other CpG islands in this region (Supplementary Figure 1). However, it is unknown whether the overlap of the intron 1 region with DNA methylation may affect the NTT-MMP-2 expression.

The NTT-MMP-2 isoform is induced by hypoxia (Lovett et al., 2012), which is present in cancer and other diseases (Ivan and Kaelin, 2017). The cells sense and adapt to hypoxia by activating hypoxia-inducible transcription factors, but the cells differ in their transcriptional response to hypoxia (Ivan and Kaelin, 2017). A probable explanation is that the hypoxia-inducible transcription factors do not bind to CpG dinucleotides that are methylated. Therefore, the specific DNA methylation patterns of a cell established under normoxic conditions determine the hypoxia-inducible binding profiles for the transcription factors, and define how cell types response to hypoxia (D’Anna et al., 2020). Taken together, these processes could explain the mechanism for regulating the latent promoter located in the intron of the *MMP2* gene in the condition of hypoxia by DNA methylation. However, these hypotheses remain to be proved.

## Functional Assessment of the NTT-MMP-2 Isoform

Cardiac-specific transgenic mice expressing the NTT-MMP-2 isoform were generated to determine its functional significance (Lovett et al., 2013). These mice developed progressive cardiomyocyte and ventricular hypertrophy associated with systolic heart failure. The NTT-MMP-2 transgenic hearts also showed more severe injury following *ex vivo* IRI. Therefore, this NTT-MMP-2 isoform induced by oxidative stress directly contributed, in the absence of superimposed injury, to cardiomyocyte hypertrophy, inflammation, systolic heart failure, and enhanced susceptibility to IRI (Lovett et al., 2013).

Further studies evaluated the effects of the presence of the NTT-MMP-2 isoform (Lovett et al., 2014). At 4 to 5-month-old transgenic mice, the NTT-MMP-2 expression was located in the mitochondria and in the Z-line of the sarcomere. When compared with wild-type mice, transgenic mice expressing the NTT-MMP-2 isoform presented impaired myocardial contraction, without decreasing myofilament force, but affecting calcium transients. However, the FL-MMP-2 impaired cardiomyocyte contractility by decreasing myofilament force. Thus, the FL-MMP-2 and NTT-MMP-2 have distinctive pathophysiological mechanisms in the cardiomyocyte by impairing different intracellular processes (Lovett et al., 2014).

Transgenic mice expressing the NTT-MMP-2 isoform specifically in the renal proximal tubule cells were generated to evaluate the effects of this isoform (Ceron et al., 2017). At 4 months of age, the NTT-MMP-2 transgenic mouse kidneys presented tubular epithelial cell necrosis, mitochondrial loss of organized cristae, and mitochondrial permeability transition, mitophagy observed at ultrastructural analysis. At 8 months old, transgenic mice expressing the NTT-MMP-2 isoform presented severe structural kidney abnormalities, as tubular atrophy, necrosis of tubular epithelial cells, and mononuclear cell infiltration and evidence of mitochondrial reactive oxygen species production. Glomerular changes were not present. At this time point, transgenic mice expressing the NTT-MMP-2 isoform also had a decrease in renal function compared with wild-type mice (Ceron et al., 2017).

## NTT-MMP-2 and Renal Dysfunction and Kidney Diseases

*Delayed graft function*, a clinical example of renal acute IRI, is a complication of renal transplantation, which results from tubular epithelial cell injury and has consequences as post-transplantation dialysis, increased incidence of acute rejection, and poorer long-term outcomes (Moeckli et al., 2019; Nieuwenhuijs-Moeke et al., 2020). The extent of tubular epithelial injury and the expression of both FL-MMP-2 and NTT-MMP-2 were analyzed in renal biopsies of controls and patients diagnosed with delayed graft function, and these expressions were correlated with the amount of tubular damage in patients (Wanga et al., 2015). While FL-MMP-2 expression was diffusely found in control kidney biopsies, NTT-MMP-2 was found located in a pattern characteristic of mitochondria only in biopsies of patients with delayed graft function (Wanga et al., 2015).

The mitochondrial NTT-MMP-2 isoform was also evaluated in *acute kidney injury*, a frequent complication in severely ill patients that may progress to *chronic kidney disease*. Mitochondria dysfunction increases oxidative stress and cause tubular inflammation, one of the major fibrotic processes in renal diseases (Maekawa and Inagi, 2019; Husain-Syed et al., 2020). Wild-type mice and transgenic NTT-MMP-2 mice were submitted to 40 min of unilateral renal IRI, and the contralateral non-clamped kidney was evaluated for systemic inflammatory responses (Ceron et al., 2017). At 96 h following IRI, the contralateral kidney of wild-type mice presented normal morphology, while the kidney subjected to IRI presented a mild degree of tubular dilatation, inflammation, and cast formation. However, the contralateral kidney of NTT-MMP-2 transgenic mice showed mild to moderate degrees of injury, and the kidneys subjected to IRI showed more extensive injury, with massive cast formation, tubular dilatation, and cellular inflammation than the wild-type kidneys subjected to IRI (Ceron et al., 2017).

Three weeks after IRI, the differences between wild-type and NTT-MMP-2 transgenic mice were more prominent. While IRI and contralateral of wild-type mice showed moderate injury, the kidney of NTT-MMP-2 mice subjected to IRI showed extensive mononuclear cell infiltration, fibrosis, and tubular epithelial cell dropout (Ceron et al., 2017). Moreover, the contralateral kidneys showed cellular inflammation, fibrosis, tubular dilatation, and tubular epithelial cell dropout, indicating a sustained systemic inflammatory response. These findings suggest that the NTT-MMP-2 sensitizes the kidneys to more severe IRI (Ceron et al., 2017).

The NTT-MMP-2 was also showed to induce sustained systemic inflammatory response after IRI, which was not observed in the wild-type kidneys. The kidney of the NTT-MMP-2 transgenic mice present enhanced expression of *OAS-1*, *IRF-7*, and *CXCL-10* at 96 h following IRI when compared with IRI kidneys of the wild-type mice and the contralateral kidneys, suggesting the induction of a systemic inflammatory response by NTT-MMP-2. This enhanced expression of innate immunity genes and a sustained release of danger-associated molecular patterns were persistent 3 weeks following the IRI in the kidney subjected to the injury and the contralateral kidney of NTT-MMP-2 transgenic mice (Ceron et al., 2017).

The NTT-MMP-2 also participates in chronic kidney disease. The HypoE/SR-B1 Mx1-Cre mice is a mice model of accelerated atherogenesis, which develops a diffuse atherosclerosis, chronic kidney disease and ischemic cardiomyopathy were given a high-fat diet (Wang et al., 2014; Luk et al., 2016). After 22 days of high-fat diet, an increased expression of both FL-MMP-2 and NTT-MMP-2 was associated with tubular epithelial cell necrosis, kidney inflammation, and elevated plasma blood urea nitrogen levels when compared with normal diet-fed mice (Ceron et al., 2017).

Normal *aging* also leads to a decline in the kidney function. While the mechanisms are not fully known, oxidative stress and inflammation may participate in the aging changes in organ functions (Panickar and Jewell, 2018). NTT-MMP-2 was increased in the renal proximal tubules in aged mouse (14 months old wild-type mice), but it was absent at 4 months old wild-type mice. No differences were observed in FL-MMP-2 expression as a function of increasing age. The NTT-MMP-2 was suggested to be a link between the inflammatory state and the declined renal function that occurs on the aging process (Ceron et al., 2017).

*Diabetic nephropathy* is a complication of diabetes mellitus and a frequent cause of chronic kidney disease. The participation of both MMP-2 isoforms was also examined in diabetic nephropathy (Kim et al., 2017). The increased expression of the FL-MMP-2 and NTT-MMP-2 was observed in HK2 cells cultured in high glucose or 4-hydroxy-2-hexenal (an oxidative stress inductor). However, the pretreatment of HK2 cells with the antioxidant/NF-κB inhibitor pyrrolidine dithiocarbamate inhibited only the NTT-MMP-2 expression. In the murine model of type 1 diabetes mellitus induced by streptozotocin, NTT-MMP-2 was intensely expressed in the diabetic kidneys, while FL-MMP-2 was present in control and diabetic kidneys. Finally, an increase in both MMP-2 isoforms was found in renal biopsies of patients with diabetic nephropathy (Kim et al., 2017).

To explore the possible mechanisms of aging in renal function, the FL-MMP-2 and NTT-MMP-2 were examined in two mouse models of chronic kidney disease, the streptozotocin-induced model of type 1 diabetes mellitus, and the 5/6 nephrectomy model of chronic kidney disease in mice aged 8 weeks (young mice) or 14 months (old mice). The expression of both isoforms was increased independently of mice age in both mouse models. However, only the NTT-MMP-2 expression was increased in mice aged 14 months, which was associated with the tubulointerstitial fibrosis development in chronic kidney disease (Rhee et al., 2018).

The temporal and spatial locations of both MMP-2 isoforms were examined in a mouse model of type 1 diabetes mellitus induced by streptozotocin and in the db/db mouse model of type 2 diabetes mellitus. Both the FL-MMP-2 and NTT-MMP-2 were increased earlier in the kidney of streptozotocin mice than in db/db mice. However, while FL-MMP-2 was located in the cortices and outer medullae, NTT-MMP-2 was located only in the cortices. Moreover, the levels of nitrotyrosine, a marker of nitrosative stress, were increased similarly to the NTT-MMP-2 isoform (Kim et al., 2018).

## NTT-MMP-2 and Heart Injury

Diabetic cardiomyopathy is a condition associated with enhanced reactive oxygen species production and mitochondrial dysfunction (Cieluch et al., 2020). The expression of FL-MMP-2 and NTT-MMP-2 was increased both in H9C2 cells exposed to high glucose media and in the heart of streptozotocin-induced diabetes mouse model. The FL-MMP-2 was located in the cardiomyocyte sarcomeres, and the NTT-MMP-2 mainly in the subsarcolemmal space, where mitochondria are abundant. The degree of mitochondrial damage was positively correlated to NTT-MMP-2 expression, and the decreased left ventricular systolic function observed in diabetic mice was associated with the increased expression of both MMP-2 isoforms (Lee H. W. et al., 2019).

Increased cardiac MMP-2 activity was found in hearts of mice treated with doxorubicin, in part, by upregulating NTT-MMP-2. Cardiotoxicity was attenuated by MMP inhibitors (Chan et al., 2021). Regarding MMPs inhibitors, they are shown to be protective in different models of renal and cardiac diseases, including type 1 diabetes (Yaras et al., 2008), *in vivo* renal injury (Labossiere et al., 2015; Lee T. F. et al., 2019), and other models of cardiac injury and cardiovascular dysfunction (Kandasamy et al., 2010; Rizzi et al., 2010; Guimaraes et al., 2011; Castro et al., 2012). In this context, FL-MMP-2 and NTT-MMP-2 are relevant targets of pharmacological intervention.

## Conclusion and Perspectives

We provide insights into the epigenetic regulation of the latent promoter located in the first intron of *MMP-2*, which support the activation of the NTT-MMP-2 isoform. Moreover, we reviewed recent evidence for the presence of NTT-MMP-2 in renal dysfunction and in kidney and cardiac diseases. Noteworthy, both the FL-MMP-2 and NTT-MMP-2 isoforms can be activated in tissue injury/oxidative stress models. However, FL-MMP-2 is directly activated by oxidative stress whereas there is transcriptional activation and expression of NTT-MMP-2. Further studies should consider that these isoforms would act in different time frames, subcellular locales, and protein targets in the development of tissue injuries and diseases. Taken together, these findings may help to understand how hypoxia and oxidative stress trigger NTT-MMP-2 expression, which are relevant pathophysiological mechanisms to several diseases. Our insights may guide further experimental and clinical studies that are needed to elucidate the underlying mechanisms and the role of NTT-MMP-2 in renal dysfunction. We expect that these future efforts may help to establish the NTT-MMP-2 as a potential therapeutic target in kidney diseases.

## Data Availability Statement

Publicly available datasets were analyzed in this study. This data can be found here: UCSC Genome Browser Gateway, available at: https://genome.ucsc.edu/cgi-bin/hgTracks?db=hg 38&lastVirtModeType=default&lastVirtModeExtraState=&virtM odeType=default&virtMode=0&non-VirtPosition=&position=ch r16%3A55478079%2D55483623&hgsid=963348143_Mp3s46Yx zZ1zGnQs8Ct0oWoyZOKp.

## Author Contributions

JC, ML, and CC drafted the manuscript and prepared figures. JC, AS, ML, and CC edited and revised the manuscript. All authors have read and approved the final version of manuscript for submission.

## Conflict of Interest

The authors declare that the research was conducted in the absence of any commercial or financial relationships that could be construed as a potential conflict of interest.

## Supplementary Material

Supplementary Figure 1 UCSC Genome Browser on Human Feb. 2009 (GRCh37/hg19) Assembly. Characterization of the genomic position chr16:55,512,747-55,517,056 showing the location of CpG dinucleotides and CpG islands located in the *MMP2* gene. **(A)** The CpG methylation by Methyl 450K Bead Array ENCODE/HAIB showing the CpG dinucleotides and CpG islands in the promoter region/exon 1 and intron 1 that are not methylated in most of the ENCODE cell lines, except in the HeLa-S3 line, and the DNA methylation by reduced representation bisulfite Seq from ENCODE/HudsonAlpha showing the same pattern of methylation in this region. **(B)** The output of MethPrimer showed other CpG dinucleotides and CpG islands in the *MMP2* gene, which are not covered by the ENCODE techniques publicly available at the UCSC Genome Browser.
