# Supplementary figures and images for: Epigenetic Regulation of the N-Terminal Truncated Isoform of Matrix Metalloproteinase-2 (NTT-MMP-2) and Its Presence in Renal and Cardiac Diseases
Source: Front Genet. 2021 Feb 25;12:637148. doi: 10.3389/fgene.2021.637148 (PMC7959838; doi:10.3389/fgene.2021.637148)

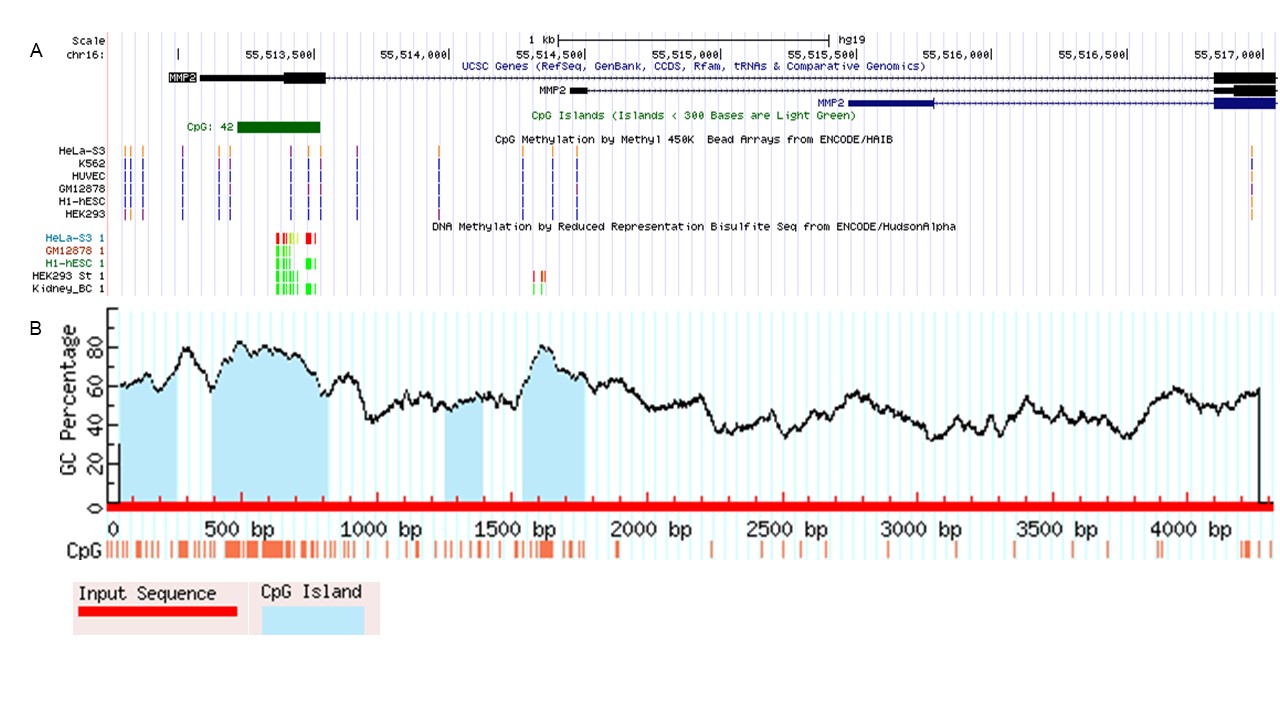

Supplement: Supplementary Figure 1 — UCSC Genome Browser on Human Feb. 2009 (GRCh37/hg19) Assembly. Characterization of the genomic position chr16:55,512,747-55,517,056 showing the location of CpG dinucleotides and CpG islands located in the MMP2 gene. (A) The CpG methylation by Methyl 450K Bead Array ENCODE/HAIB showing the CpG dinucleotides and CpG islands in the promoter region/exon 1 and intron 1 that are not methylated in most of the ENCODE cell lines, except in the HeLa-S3 line, and the DNA methylation by reduced representation bisulfite Seq from ENCODE/HudsonAlpha showing the same pattern of methylation in this region. (B) The output of MethPrimer showed other CpG dinucleotides and CpG islands in the MMP2 gene, which are not covered by the ENCODE techniques publicly available at the UCSC Genome Browser. [file Image_1.JPEG]
