# Supplementary material for: Epigenetic Regulation of the N-Terminal Truncated Isoform of Matrix Metalloproteinase-2 (NTT-MMP-2) and Its Presence in Renal and Cardiac Diseases
Source: Front Genet. 2021 Feb 25;12:637148. doi: 10.3389/fgene.2021.637148 (PMC7959838; doi:10.3389/fgene.2021.637148)
Supplement: Supplementary file 3 [file Data_Sheet_1.docx]

# SUPPLEMENTARY MATERIAL

**SUPPLEMENTARY FIGURE 1 LEGEND**

**Supplementary Figure 1:** UCSC Genome Browser on Human Feb. 2009 (GRCh37/hg19) Assembly. Characterization of the genomic position chr16:55,512,747-55,517,056 showing the location of CpG dinucleotides and CpG islands located in the *MMP-2* gene. **A)** The CpG Methylation by Methyl 450k Bead Array ENCODE/HAIB showing the CpG dinucleotides and CpG islands in the promoter region/exon 1 and intron 1 that are not methylated in most of the ENCODE cell lines, except in the HeLa-S3 line, and the DNA Methylation by Reduced Representation Bisulfite Seq from ENCODE/HudsonAlpha showing the same pattern of methylation in this region. **B)** The output of MethPrimer showed others CpG dinucleotides and CpG islands in the *MMP-2* gene, which are not covered by the ENCODE techniques publicly available at the UCSC Genome Browser.
